# Regulation of IL-10 production in dendritic cells is controlled by the co-activation of TLR2 and Mincle by *Lactiplantibacillus plantarum* OLL2712

**DOI:** 10.1128/spectrum.01196-24

**Published:** 2025-02-04

**Authors:** Ryuhei Sakabe, Kazumasa Onishi, Junko Mochizuki, Takayuki Toshimitsu, Tomoyuki Shimazu, Shigenobu Kishino, Jun Ogawa, Sho Yamasaki, Toshihiro Sashihara

**Affiliations:** 1 Food Microbiology and Function Research Laboratories, R&D Division, Meiji Co., Ltd., Hachioji, Tokyo, Japan; 2 Fermentation Development Research Department Food Development Laboratories, R&D Division, Meiji Co., Ltd., Hachioji, Tokyo, Japan; 3 Division of Applied Life Sciences, Graduate School of Agriculture, Kyoto University, Kyoto, Japan; 4 Department of Molecular Immunology, Research Institute for Microbial Diseases, Osaka University, Suita, Osaka, Japan; 5 Laboratory of Molecular Immunology, Immunology Frontier Research Center (IFReC), Osaka University, Suita, Osaka, Japan; Geisel School of Medicine at Dartmouth, Lebanon, New Hampshire, USA

**Keywords:** lactic acid bacteria, interleukin-10, anti-inflammation, Toll-like receptor 2, macrophage inducible C-type lectin receptor, phagocytosis

## Abstract

We showed that *Lactiplantibacillus plantarum* OLL2712 (OLL2712) strongly induces interleukin (IL)-10 production in immune cells. Although beneficial effects of this strain have been observed in both mice and humans, the mechanisms underlying IL-10 induction remain unclear. In this study, we found that OLL2712 co-activates two pattern recognition receptors, leading to IL-10 production in the mouse-derived thermosensitive dendritic cell line, tsDC. We first revealed the involvement of the Toll-like receptor (TLR)2-Myeloid differentiation primary response gene (MYD) 88 pathway in OLL2712-induced IL-10 production in tsDCs. However, stimulation with the TLR2 agonist alone was insufficient to induce IL-10 production. Consequently, we explored additional signaling pathways and found that the phosphorylation of spleen tyrosine kinase (Syk) was important in response to OLL2712, which was not triggered by a TLR2 agonist alone. Notably, the activation of Syk was found to depend on macrophage-inducible C-type lectin receptor (Mincle), one of the C-type lectin receptors. However, the surface-expressed Mincle is not responsible for the IL-10 production by OLL2712. Instead, it depends on the incorporation of OLL2712 into tsDCs, suggesting that Mincle recognizes incorporated OLL2712 intracellularly. In summary, OLL2712 is initially recognized by TLR2, which subsequently induces the expression of Mincle to recognize incorporated OLL2712, ultimately inducing IL-10 production.

**IMPORTANCE:**

The objective of this study is to elucidate the mechanism by which *Lactiplantibacillus plantarum* OLL2712 (OLL2712), previously identified by our research group as a potent stimulator of interleukin-10 production in immune cells, exerts its immunomodulatory effects. Our findings indicate that OLL2712 acts in synergy with two pattern-recognition receptors: Toll-like receptor 2 and Macrophage inducible C-type lectin receptor (Mincle). Additionally, we observed that OLL2712 needs to be internalized intracellularly to be recognized by Mincle. These findings represent the first insights into the detailed mechanism underlying the anti-inflammatory effects of OLL2712.

## INTRODUCTION

Some lactic acid bacteria (LAB) have been reported to modulate immune activity and prevent or ameliorate immune-related disorders, including inflammatory bowel diseases, allergies, and certain metabolic disorders (1–3). Specific LAB strains have been selected for their efficacy in treating these disorders (4–6). Active research on such applications is likely due to the general acceptance of LAB by the consumers, based on their long dietary experience. Therefore, if further detailed studies on LAB reveal novel applications with stronger effects, they could lead to the development of easily accessible and effective foods for many individuals.

Interleukin (IL)-10 is an immunosuppressive factor that plays a vital role in regulating inflammation and is primarily secreted by dendritic cells and macrophages (7). Several studies have reported that LAB that induce IL-10 production can ameliorate several immune-related disorders. For example, Hu et al. reported that *Lactobacillus murinus* promotes IL-10 release from macrophages and reduces ischemia/reperfusion injury in mouse intestines (8). Jia et al. reported that *L. johnsonii* promotes IL-10 production via macrophages and suppresses colitis in dextran sulfate sodium-induced mice (9). These reports indicate that the high IL-10 production ability of LAB may have promising health effects.

In our previous study, we identified a LAB strain, *Lactiplantibacillus plantarum* OLL2712 (formerly reported as *Lactobacillus plantarum* OLL2712), with a robust capability to induce IL-10 via TLR2-MYD88 signaling pathway in mouse bone marrow- and intestine-derived dendritic cells (BMDCs) (10–12). Oral administration of heat-treated OLL2712 cells alleviated chronic inflammation and improved glucose and lipid metabolism in obesity and diabetes mouse models (10, 12, 13). Furthermore, ingestion of heat-treated OLL2712 cells not only reduced body fat accumulation, deterioration of glycemic control, and chronic inflammation in healthy and overweight human adults (14–17) but also had protective effects against memory function decline in older adults (18). In this way, we demonstrated that LAB with a strong IL-10 induction capability also have positive effects on human health.

Although numerous reports, including ours, have highlighted the beneficial effects of LAB on the host, the specific mechanisms underlying the robust induction of IL-10 production by these LAB strains remain unclear. Our study aimed to elucidate the IL-10-inducing mechanisms of OLL2712 in immune cells and its underlying physiological effects. To elucidate the mechanism of IL-10 induction, we employed a conditionally immortalized, thermosensitive dendritic cell line, tsDC as a suitable alternative to BMDCs, considering animal welfare.

## MATERIALS AND METHODS

### Bacterial strain and growth conditions

*L. plantarum* OLL2712 (deposit no. FERM BP-11262), isolated in our laboratory as described previously (10), was cultured in de Man, Rogosa, and Sharpe (MRS) broth (Becton Dickinson, Cockeysville, MD, USA) at 37°C for 18 h. Cultured OLL2712 cells were washed twice with phosphate-buffered saline (PBS) pH 7.2 and twice with distilled water. The cells were suspended in distilled water and heat-treated at 75°C for 1 h. The lyophilized cells were resuspended in PBS for *in vitro* studies.

### tsDC culture

tsDCs were purchased from the European Collection of Authenticated Cell Cultures (Salisbury, UK) and cultured in Iscove’s Medium with GlutaMAX Supplement (Invitrogen, Carlsbad, CA, USA) containing 5% fetal bovine serum , 2 mM glutamine, 100 U/mL penicillin, 100 µg/mL streptomycin, and 0.1% 2-mercaptoethanol at 33°C in 9% CO_2_.

### Extraction of lipoteichoic acid (LTA) from OLL2712

LTA was prepared from OLL2712 by using the method described by Suda et al. (19). Briefly, OLL2712 cells were disrupted through sonication. Crude LTA was extracted using hot phenol and treated with nuclease. LTA was purified via column chromatography using Octyl Sepharose CL-4B (Cytiva, Marlborough, MA, USA).

### IL-10 production assay

The tsDCs were plated in 96-well plates (1.0 × 10^5^ cells/well) and stimulated with OLL2712, Pam3CSK4 (Invitrogen), LTA derived from OLL2712 (OLL2712 LTA), trehalose-6,6-dimycolate (TDM), and trehalose-6,6-dibehenate (TDB) (Invitrogen) for 24 h. The IL-10 levels in the supernatant were measured using an IL-10 enzyme-linked immunosorbent assay set (BD Biosciences, Franklin Lakes, NJ, USA) according to the manufacturer’s instructions. TDM was dissolved in isopropanol and added to the wells, followed by the evaporation of the solvent before cell treatment.

### Chemical inhibitors and neutralization antibodies

To inhibit the phosphorylation of spleen tyrosine kinase (Syk), endocytosis, or autophagosome-lysosome fusion, tsDCs were pre-treated for 1 h with 10 µM piceatannol (Abcam, Cambridge, UK), 10 µM dynasore (Abcam), or 10 nM bafilomycin A1 (Sigma-Aldrich, St. Louis, MO, USA), respectively.

For neutralization of Toll-like receptor (TLR)2 or Mincle, tsDCs were pre-treated for 1 h with 3 µg/mL anti-TLR2 antibody (Thermo Fisher Scientific, Waltham, MA, USA) or 3 µg/mL anti-Mincle antibody (Thermo Fisher Scientific), respectively. Each isotype was used as the control.

### Knockdown assay

tsDCs were plated in 12-well plates (5.0 × 10^5^ cells/well) and transfected with 10 nM Silencer Select pre-designed small interfering RNAs (siRNAs; Thermo Fisher Scientific) targeting Myeloid differentiation primary response gene (MYD)88, Fc receptor gamma (FcRγ), Mincle, Dectin-2, and their control via electroporation using a Neon Transfection System (Thermo Fisher Scientific), according to the manufacturer’s instructions.

### Quantitative real-time PCR

Total RNA was extracted using the Maxwell RSC simplyRNA Cells Kit (Promega, Madison, WI, USA), following the manufacturer’s instructions. Purified RNA was reverse transcribed using the PrimeScript RT Master Mix (Takara Bio, Shiga, Japan). Subsequently, quantitative real-time PCR (qPCR) was performed using SYBR Premix Ex Taq II (Takara Bio) and Quantstudio 3 (Thermo Fisher Scientific). Mean fold changes were analyzed using the relative standard curve method. The primers used in this study are listed in Table 1. The PCR cycle conditions were as follows: two-step cycling, 95°C for 30 s; 40 cycles of 95°C for 5 s; and 60°C for 30 s. Data were normalized to the values of glyceraldehyde-3-phosphate dehydrogenase gene (*Gapdh*), and the results were expressed as fold-changes to relative controls.

**TABLE 1:** Primer sequences used for quantitative real-time PCR

Gene	Direction	Sequence (5′→3′)
*Gapdh*	Forward	CAGAACTACATCCCTGCATCC
	Reverse	CCACCTTCCTGATGTCATCA
*Clec4e*	Forward	CAGTGGCAATGGGTGGATGATAC
	Reverse	AGTCCCTTATGGTGGCACAGTC
*Clec4n*	Forward	GAACCACAAGCCCACAGAATC
	Reverse	TCTTCCCATGCATGATCCAA
*Myd88*	Forward	CATACCCTTGGTCGCGCTTA
	Reverse	CCAGGCATCCAACAAACTGC

### Immunoblotting

The cells were lysed with radioimmunoprecipitation buffer (Nacalai Tesque, Kyoto, Japan) containing a protease inhibitor cocktail (Sigma-Aldrich) and a phosphatase inhibitor (Sigma-Aldrich). Cell lysates were treated with Laemmli sample buffer (Bio-Rad, Hercules, CA, USA) containing 50 mM dithiothreitol, boiled for 5 min, separated using sodium dodecyl sulfate-polyacrylamide gel electrophoresis and transferred onto polyvinylidene difluoride membranes (Bio-Rad). The membrane was blocked with Every Blot blocking buffer (Bio-Rad) for 5 min. After the membrane was washed three times with PBS containing 0.05% Tween-20, a primary rat monoclonal anti-Mincle (MBL, Tokyo, Japan), rabbit monoclonal anti-p-Syk (Thermo Fisher Scientific), rabbit polyclonal anti-Syk (Thermo Fisher Scientific), rabbit monoclonal anti-IκBα (Cell Signaling Technology, Danvers, MA, USA), rabbit monoclonal anti-MYD88 (Abcam), or anti-beta actin antibody (Abcam) was added to the membrane in the blocking buffer and incubated at room temperature (RT) for 1 h. As secondary antibodies, anti-rat or rabbit immunoglobulin (Ig)G antibodies conjugated with horseradish peroxidase (Cell Signaling Technology) were incubated with the membrane in blocking buffer at RT for 1 h. Images of the luminescent signals were acquired using a ChemiDoc (Bio-Rad) with an ECL plus reagent (Cytiva).

### OLL2712 labeling and confocal microscopy

Lyophilized OLL2712 (1 mg) was resuspended in 100 µL carbonate buffer (pH 8.3) containing 1 µg Alexa Fluor 488 carboxylic acid, succinimidyl ester (Invitrogen), and incubated at 37°C for 1 h. After two washes with PBS, the cells were incubated with Alexa Fluor 488-labeled OLL2712. tsDCs were stained with Cell Mask Deep Red (Thermo Fisher Scientific) and observed using an LSM 880 confocal microscope (Carl Zeiss, Jena, Germany).

### Flow cytometry

For phagocytosis assay, tsDCs were incubated in the presence of 100 µg/mL Alex Fluor 488-labeled OLL2712 for 24 h. To examine Dectin-2 expression, tsDCs were stained with Dectin-2 antibody (R&D Systems, Minneapolis, MN, USA) and detected with APC-conjugated anti-goat IgG (R&D systems). Cells were harvested and labeled with 7-amino-actinomycin D (7-AAD; DC Biosciences, Dundee, Scotland, UK). The tsDCs were washed twice with PBS, and 7-AAD-negative cells were excluded using a flow cytometer FACSVerse (Becton Dickinson).

### Cell viability assay

tsDCs were plated in 96-well plates at a density of 1.0 × 10^5^ cells/well. After 24 h, cell viability was measured using a WST-1 cell viability assay kit (Takara Bio).

### Statistical analysis

Statistical analyses were performed using GraphPad Prism 8 (GraphPad Software, San Diego, CA, USA). Comparisons between two groups were evaluated using an unpaired two-tailed Student’s *t* test, and differences among multiple groups were evaluated using Tukey’s multiple comparison test. Time course of mRNA expression was evaluated using the Holm–Sidak test. Differences were considered significant at *P* < 0.05.

## RESULTS

### TLR2-MYD88 signaling is necessary for OLL2712-induced IL-10 production in tsDCs

**Fig 1:**
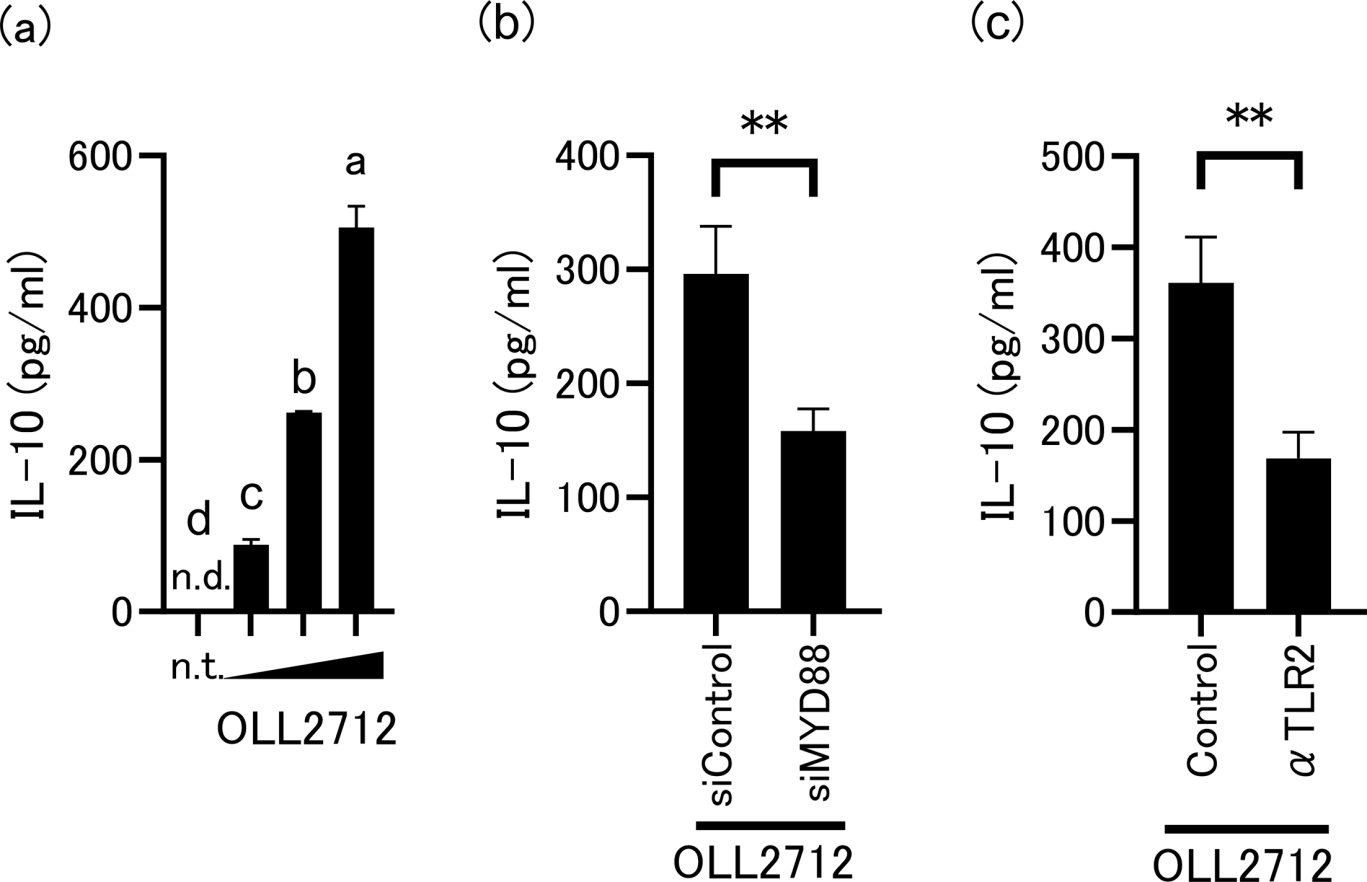
(**a**) Effects of OLL2712 treatment on interleukin (IL)-10 production in thermosensitive dendritic cells (tsDCs). tsDCs were cultured with various concentrations of OLL2712 (50, 100, and 200 µg/mL) for 24 h. n.d. indicates not detected. n.t. indicates not treated for tsDCs. Values not sharing a common letter are significantly different (*P* < 0.01). The lower detection limit of IL-10 in this method is 31.3 µg/mL. Effects of (**b**) *Myd88* knockdown or (**c**) Toll-like receptor (TLR)2 neutralization on OLL2712-induced IL-10 production. tsDCs were electroporated with small interfering (si)RNA targeting *Myd88* (siMYD88) or the control siRNA (siControl), followed by OLL2712 stimulation. tsDCs were pre-treated with anti-TLR2 antibody or the isotype control for 1 h, followed by OLL2712 stimulation (100 µg/mL). Data are shown as the mean ± SD of cultured wells (*n* = 3). **P < 0.01.

We initially examined whether activation of tsDCs by OLL2712 induces IL-10 production. As shown in Fig. 1a, OLL2712 stimulated IL-10 production in a dose-dependent manner. Since our previous observation showed that IL-10 production was not induced by OLL2712 stimulation in BMDCs derived from both *TLR2*^−/−^ and *Myd88*^−/−^ mice (12), we investigated the potential role of the TLR2-MYD88 pathway in the production of IL-10 in tsDCs. We performed knockdown experiments on MYD88 gene using siRNA. The results showed that this treatment suppressed MYD88 mRNA and protein expression levels (Fig. S1a and b), leading to decreased IL-10 production from tsDCs stimulated with OLL2712 (Fig. 1b). Similarly, considerably less IL-10 production from tsDCs stimulated with OLL2712 was observed in response to anti-TLR2 neutralization antibody (Fig. 1c). We confirmed that these treatments certainly suppress the inflammatory response (IL-6) induced by Pam3CSK4, a synthesized TLR2 ligand (Fig. S1c and d), indicating their validity. These findings, similar to those described in our previous study (12), indicate that OLL2712 stimulates IL-10 production in tsDCs by activating the TLR2-MYD88 signaling pathway.

### Syk-dependent receptor is involved in OLL2712-induced IL-10 production

**Fig 2:**
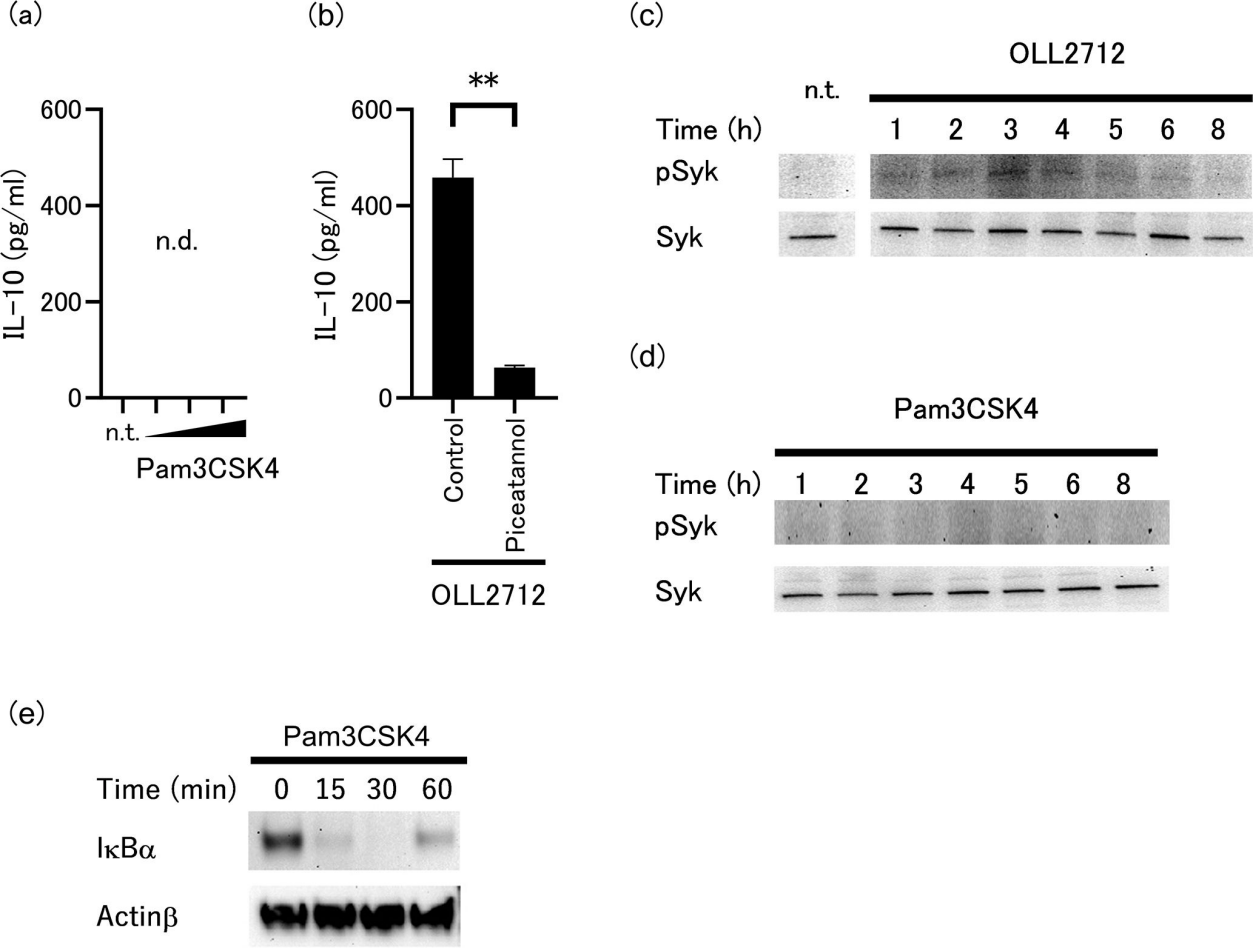
(**a**) Effect of Pam3CSK4 treatment on IL-10 production in tsDCs. tsDCs were cultured with various concentrations of Pam3CSK4 (1, 5, and 10 µg/mL) for 24 h. The lower detection limit of IL-10 in this method was 31.3 µg/mL. (**b**) Effect of piceatannol as an inhibitor of spleen tyrosine kinase (Syk) phosphorylation on OLL2712-induced IL-10 production. tsDCs were pre-treated with piceatannol for 1 h, followed by OLL2712 stimulation. Data are shown as the mean ± SD of cultured wells (*n* = 3). **P < 0.01. Immunoblotting of phosphorylated or total Syk in tsDCs stimulated by (**c**) OLL2712, or (**d**) Pam3CSK4 at indicated time points. n.t. indicates not treated for tsDCs. (**e**) Immunoblotting of IκB-α and Actinβ of tsDCs stimulated by Pam3CSK4 at indicated time points. Similar results were obtained in two experiments.

Next, we investigated whether IL-10 production was induced by Pam3CSK4 alone. As shown in Fig. 2a, there was no activity of Pam3CSK4 to produce IL-10 in tsDCs. In contrast, it could induce IL-6 in a dose-dependent manner (Fig. S2), indicating that Pam3CSK4 stimulated tsDCs. Several studies have suggested that TLR2 and C-type lectin receptor (CLR) work together to induce IL-10 (20, 21). Therefore, using piceatannol, a selective phosphorylation inhibitor of Syk, we attempted to inhibit Syk phosphorylation in tsDCs following OLL2712 stimulation to confirm the involvement of the CLR-Syk signaling pathway. The Syk inhibitor significantly reduced the production of IL-10, as shown in Fig. 2b. We confirmed that tsDC viability was unaffected by Piceatannol treatment (Fig. S3a). Immunoblotting analysis revealed that OLL2712 phosphorylated Syk after 1 h of stimulation (Fig. 2c). In contrast, Pam3CSK4 did not induce Syk phosphorylation during an 8-h period (Fig. 2d), while this treatment activated NF-κB, as indicated by the degradation of NF-κB inhibitor α (IκBα) (Fig. 2e). These findings suggest that OLL2712 can activate some types of CLRs and that OLL2712-induced IL-10 production requires Syk phosphorylation.

### Mincle plays crucial role for OLL2712-induced production of IL-10

**Fig 3:**
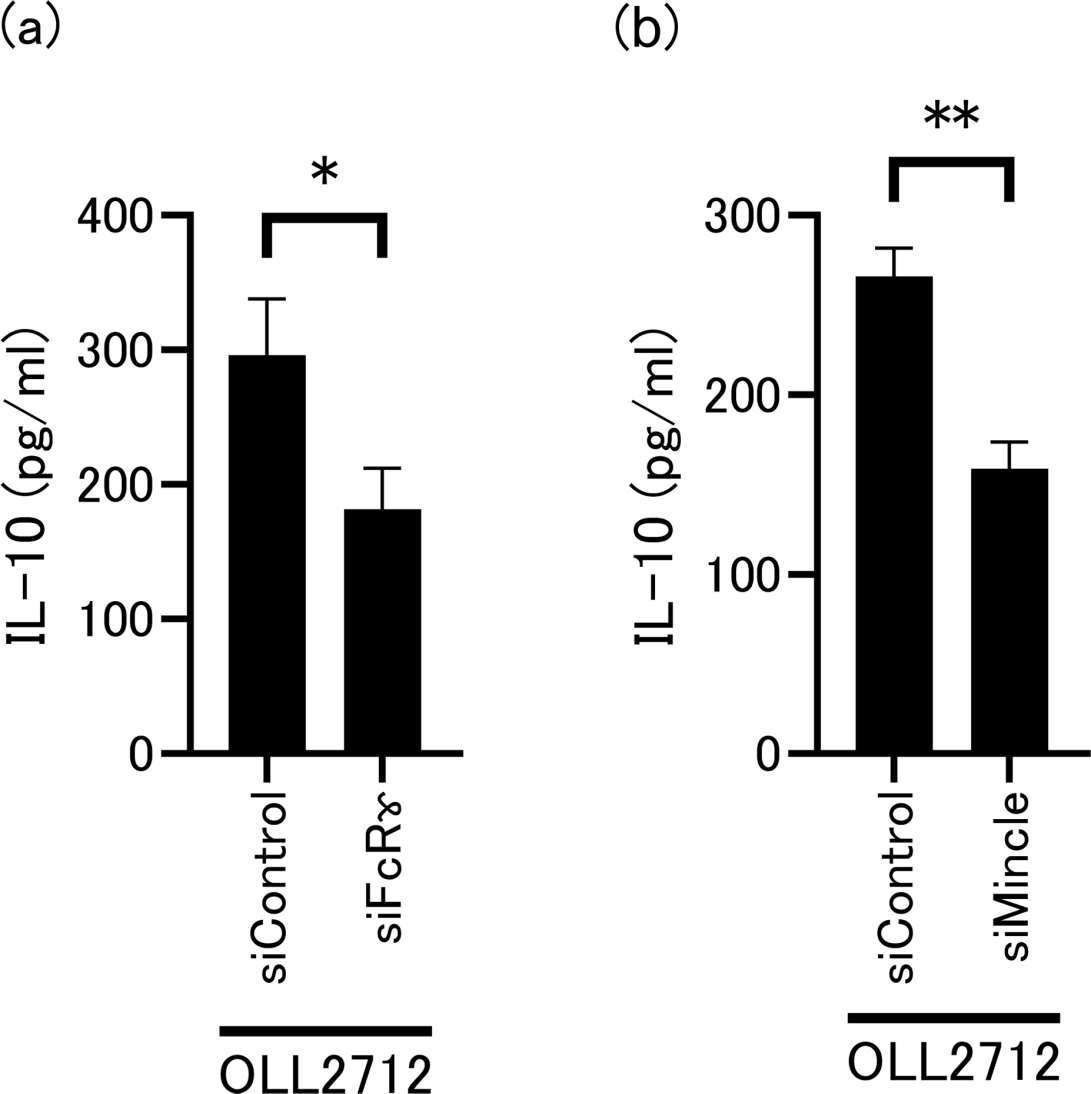
Effect of (**a**) Fc-receptor gamma (FcRɤ) or (**b**) Mincle knockdown on OLL2712-induced IL-10 production. tsDCs were electroporated with small interfering (si)RNA targeting *Fcer1g* (siFcRɤ), *Clec4e* (siMincle), or control siRNA (siControl), followed by OLL2712 stimulation. Data are shown as the mean ± SD of cultured wells (*n* = 3). *P < 0.05, **P < 0.01.

To narrow down the CLR targets involved in the induction of IL-10, we knocked down the FcRɤ, which is associated with several CLRs, such as Mincle and Dectin-2, and activates Syk. Knockdown of FcRɤ gene (*Fcer1g*) suppressed the induction of IL-10 production, as shown in Fig. 3a. Based on this finding, Mincle and Dectin-2 gene (*Clec4e* and *Clec4n*, respectively) knockdown experiments were performed to determine whether these receptors were involved in induction. IL-10 production was suppressed when *Clec4e* was knocked down (Fig. 3b). However, when *Clec4n* was knocked down, this reduction was not observed (Fig. S4a), although reduced expression of Dectin-2 mRNA and protein was observed (Fig. S4b and c). These findings suggest that Mincle is a critical receptor for triggering the production of IL-10 in tsDCs stimulated with OLL2712.

### Mincle is rapidly induced by OLL2712 stimulation

**Fig 4:**
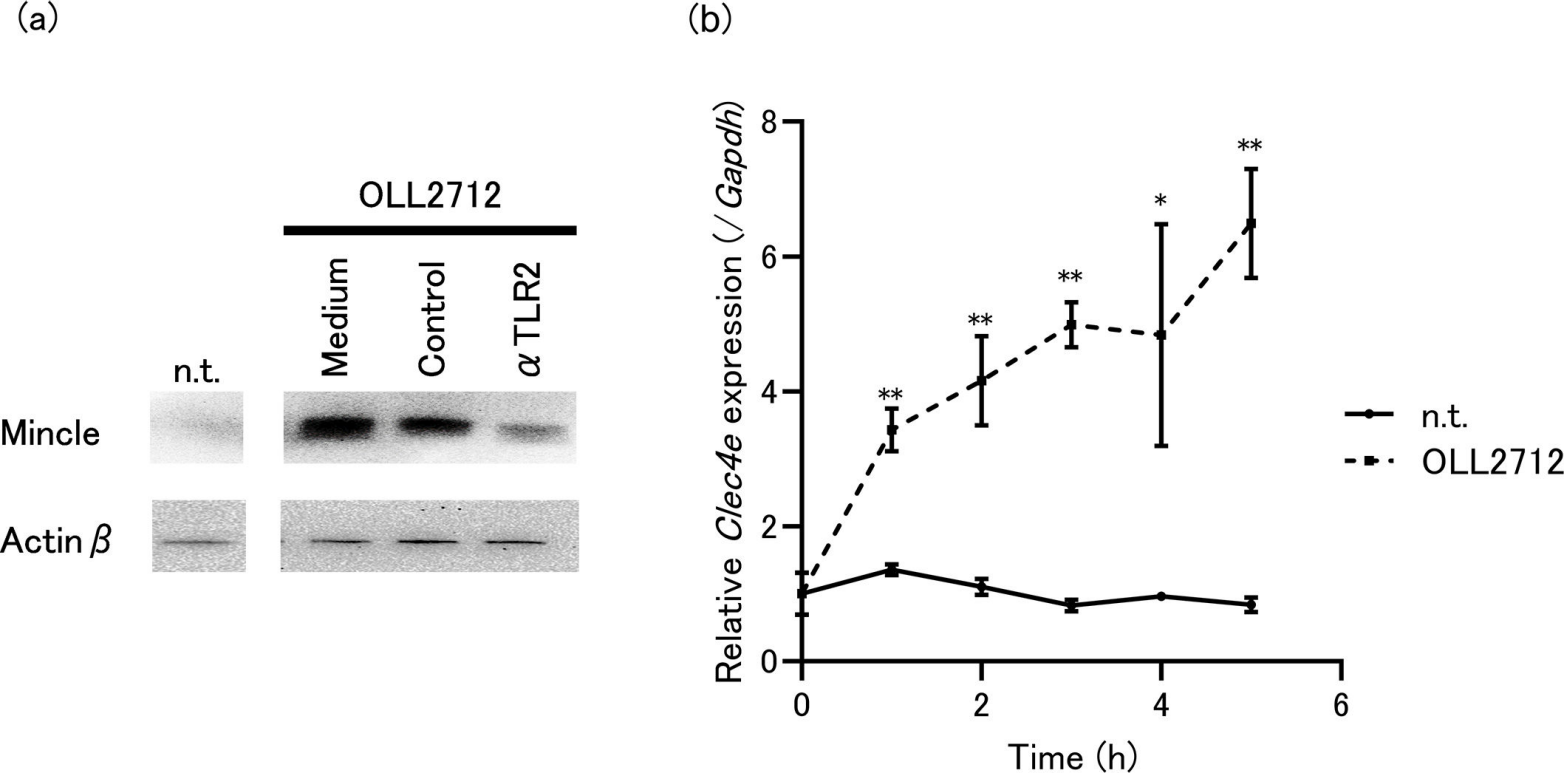
(**a**) Immunoblotting of Mincle and Actinβ of tsDCs stimulated by OLL2712 under the presence of anti-TLR2 or isotype control antibody for 24 h. n.t. indicates not treated for tsDCs. Immunoblotting experiments were repeated two times with similar results. (**b**) *Clec4e* expression levels in tsDCs stimulated with OLL2712. Relative gene expression levels were normalized to glyceraldehyde-3-phosphate dehydrogenase gene (*Gapdh*). n.t. indicates not treated for tsDCs. Data are shown as the mean ± SD of cultured wells (*n* = 3). *P < 0.05, **P < 0.01 vs n.t.

We examined the expression of Mincle under unstimulated and OLL2712-stimulated conditions. Mincle were barely expressed under unstimulated conditions but increased strongly under OLL2712-stimulated conditions, as shown by immunoblot analysis (Fig. 4a). Interestingly, after 1 h in the presence of OLL2712, Mincle gene (*Clec4e*) expression increased more than twofold and continued to increase in a time-dependent manner for up to 5 h (Fig. 4b). In contrast, Dectin-2 gene (*Clec4n*) expression remained unchanged compared to control levels (Fig. S5). These findings suggest that OLL2712 specifically induces Mincle expression.

### TLR2 activation is required for Mincle expression, and LTA is involved in the induction

**Fig 5:**
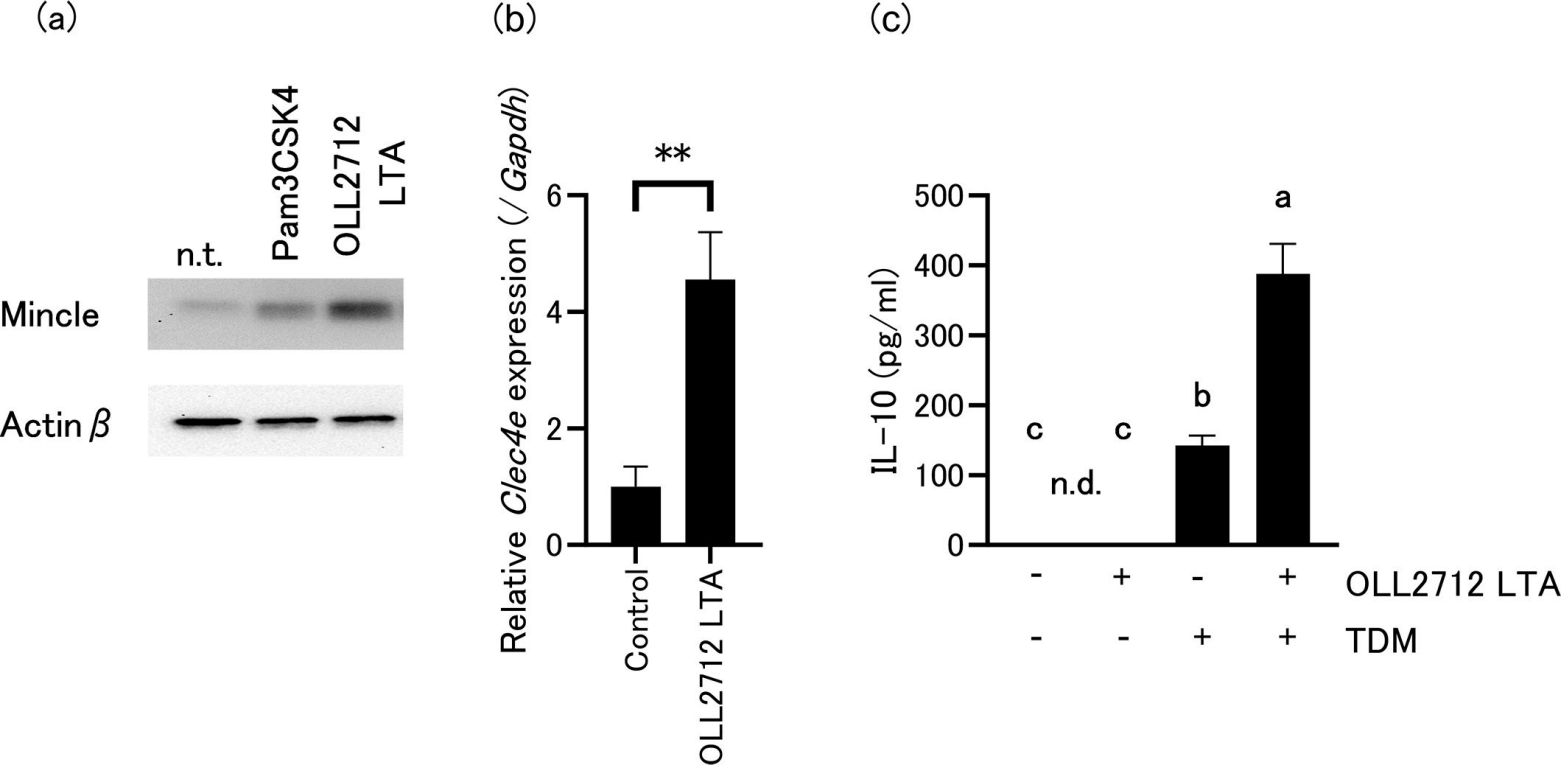
(**a**) Immunoblotting of Mincle and Actinβ of tsDCs stimulated by Pam3CSK4 or lipoteichoic acid derived from OLL2712 (OLL2712 LTA) (10 µg/mL each) for 24 h. n.t. indicates not treated for tsDCs. Immunoblotting experiments were repeated two times with similar results. (**b**) *Clec4e* expression in tsDCs stimulated with OLL2712 LTA. Relative gene expression levels were normalized to *Gapdh*. **P < 0.01. (**c**) Effect of co-stimulation with OLL2712 LTA and trehalose 6,6′-dimycolate (TDM) on IL-10 production in tsDCs. tsDCs were treated with indicated agonist and/or ligand for 24 h. The lower detection limit of IL-10 in this method is 31.3 µg/mL. Values not sharing a common letter are significantly different (*P* < 0.01). Data are shown as the mean ± SD of cultured wells (*n* = 3).

To identify the relationship between TLR2 and Mincle induction in the deduced mechanism, we pre-treated OLL2712-stimulated cells with an anti-TLR2 antibody. As shown in Fig. 4a, Mincle expression decreased compared to that in the isotype control. These data indicate that Mincle can be induced by TLR2 signaling. LTA is a well-known TLR2 ligand derived from gram-positive bacteria such as LAB (22). Therefore, we examined whether OLL2712 LTA can induce Mincle expression. Immunoblotting revealed that OLL2712 LTA induced Mincle expression within 24 h, similar to Pam3CSK4 (Fig. 5a). Moreover, qPCR revealed that OLL2712 LTA upregulated *Clec4e* expression within 24 h (Fig. 5b). To examine whether cooperative activation of TLR2 and Mincle increases IL-10 production, we used OLL2712 LTA as a ligand for TLR2 and TDM as a ligand for Mincle. As shown in Fig. 5c, stimulation with OLL2712 LTA alone did not induce IL-10 production. In contrast, TDM alone induced IL-10 production, and co-stimulation with OLL2712 LTA resulted in a stronger induction of IL-10 production. These data suggest that LTA is responsible for activating TLR2 in OLL2712-induced IL-10 and validate the collaborative mechanism between TLR2 and Mincle in inducing IL-10 production in tsDCs.

### Phagocytosis and digestion are associated with IL-10 induction by OLL2712

**Fig 6:**
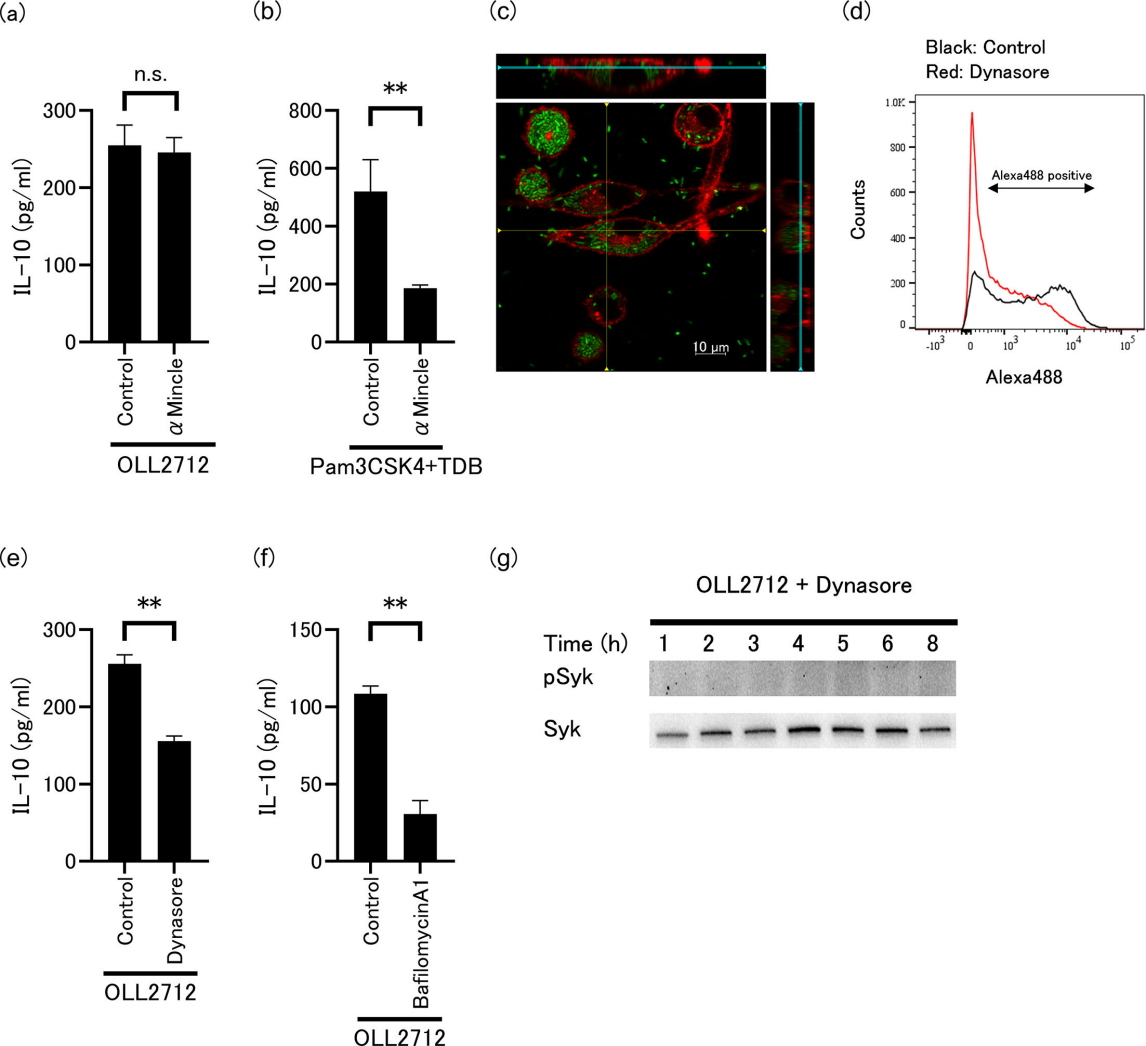
Effect of anti-Mincle antibody on (**a**) OLL2712 or (**b**) Pam3CSK4 and trehalose 6,6′-dibehenate (TDB)-induced IL-10 production. tsDCs were pre-treated with anti-Mincle antibody or its isotype control for 1 h. n.s. indicates no significance. Data are shown as the mean ± SD of cultured wells (*n* = 3). **P < 0.01. (**c**) Image of OLL2712 incorporation by tsDCs. Cell Mask labeled tsDCs (red) were incubated with Alexa Flour 488-labeled OLL2712 (green) for 24 h. The right and upper panels show *x*-*z* and *y*-*z* stacks at yellow lines. Light blue lines indicate *z*-stacks of main panel. The cells were observed using confocal laser scanning microscopy. (**d**) Flow cytometric analysis of the inhibition of phagocytosis using dynasore. tsDCs were incubated with Alexa Flour 488-labeled OLL2712 for 24 h. Effect of inhibition on phagocytosis or lysosome digestion using (**e**) dynasore or (**f**) bafilomycin A1 on OLL2712-induced IL-10 production. tsDCs were pre-treated with either dynasore or bafilomycin A1, or its solvent (dimethyl sulfoxide; DMSO) for 1 h, followed by IL-10 assay. Data are shown as the mean ± SD of cultured wells (*n* = 3). **P < 0.01. (**g**) Immunoblotting of phosphorylated Syk and total Syk in tsDCs stimulated by OLL2712 in the presence of dynasore. Immunoblotting experiments were repeated two times with similar results.

We then investigated the effect of anti-Mincle antibody on OLL2712-induced IL-10 production to evaluate whether Mincle activation was involved in this mechanism. Interestingly, we found that pre-treatment of tsDCs with the anti-Mincle antibody did not affect IL-10 production (Fig. 6a). Notably, we confirmed that this antibody suppressed IL-10 production upon co-stimulation with Pam3CSK4 and TDB, a well-known Mincle ligand analog (23) (Fig. 6b). These phenomena raised the question of how OLL2712 interacts with Mincle. This suggests that OLL2712 does not interact with the Mincle expressed on the surface of tsDCs. Therefore, we used confocal microscopy to clarify whether OLL2712 was incorporated into tsDCs. The results showed that Alexa Fluor 488-labeled OLL2712 was localized inside but not on the surfaces of several tsDCs (Fig. 6c). We next investigated whether the phagocytosis of OLL2712 into tsDCs and digestion of OLL2712 in tsDCs were crucial for inducing IL-10 production. We used dynasore and bafilomycin A1. Dynasore is a potent inhibitor of the endocytic pathway by blocking the formation of coated vesicle formation (24). Bafilomycin A1 prevents the maturation of autophagic vacuoles by inhibiting autophagosome-lysosome fusion (25). We confirmed that the pre-treatment of dynasore inhibited OLL2712 incorporation (Fig. 6d). Furthermore, both dynasore and bafilomycin A1 significantly suppressed the OLL2712-induced IL-10 production (Fig. 6e and f). We confirmed that tsDCs viability was not affected by treatment with dynasore and bafilomycin A1 (Fig. S3b and c). Moreover, although OLL2712 induced the phosphorylation of Syk in tsDCs (Fig. 2c), treatment with dynasore inhibited it (Fig. 6g). These results suggested that Mincle interacts with digested OLL2712 or its fragments in tsDCs. Taken together, these results indicate that OLL2712 is recognized by Mincle in the cell after phagocytosis and digestion.

## DISCUSSION

**Fig 7:**
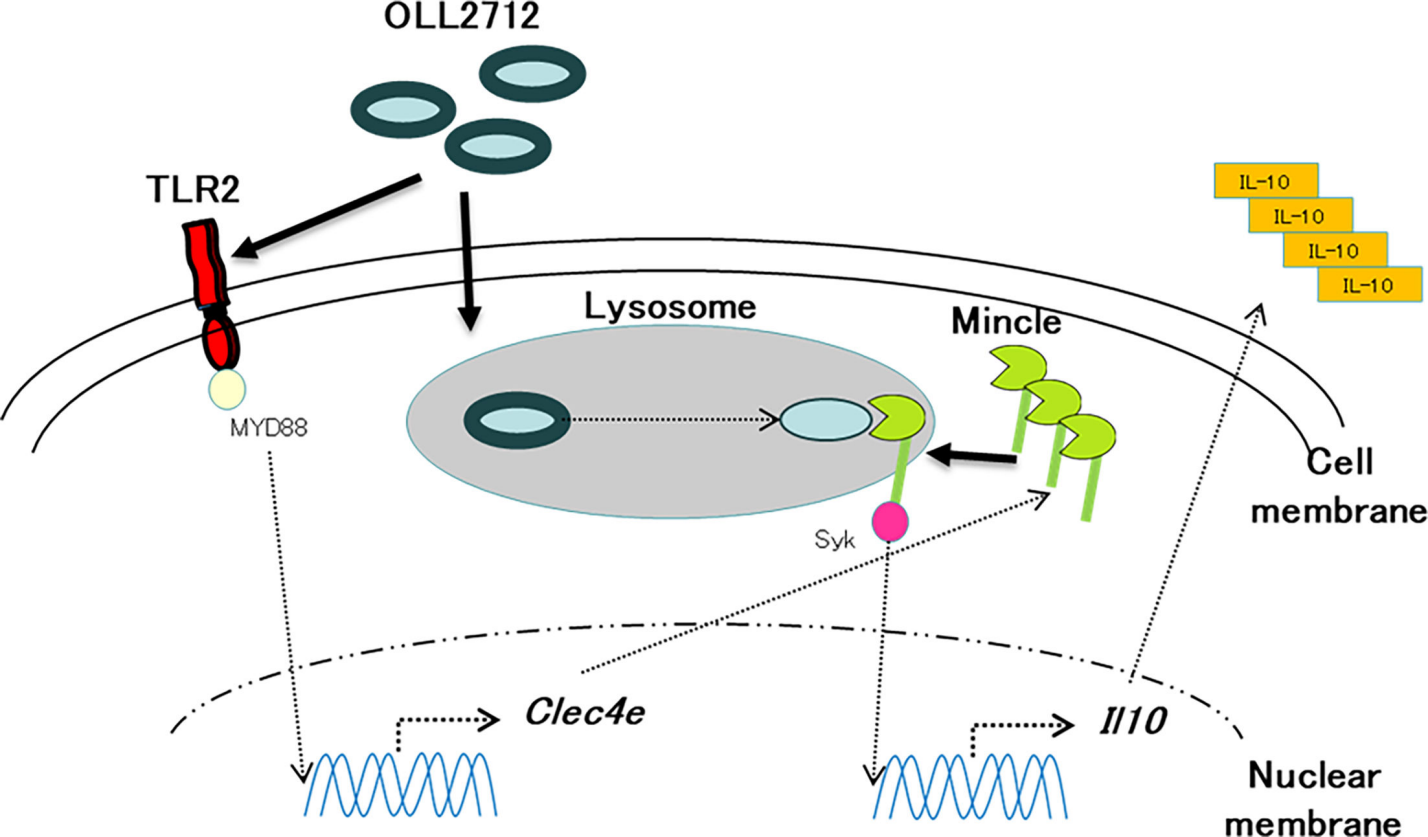
Schematic depicting the mechanism of OLL2712-induced IL-10 production by tsDCs. When OLL2712 interacts with TLR2, this interaction triggers the upregulation of Mincle expression. Subsequently, after OLL2712 is incorporated into tsDCs and digested in lysosomes, Mincle interacts with digested OLL2712 or its fragments to induce IL-10 production.

In the present study, we elucidated the underlying mechanism governing the induction of IL-10 production by OLL2712 in the conditionally immortalized dendritic cell line tsDC. Our findings highlight the indispensable role of TLR2 activation in OLL2712-induced IL-10 production within these cells. Furthermore, we found the involvement of an additional signaling pathway, ultimately identifying Syk and Mincle as pivotal components of this mechanism (Fig. 7).

Previous studies conducted in our laboratory revealed that the induction of IL-10 production in BMDCs by OLL2712 was dependent on the activation level of TLR2, a recognized regulator of IL-10 production in immune cells (12). Our present investigations with tsDCs consistently supported the TLR2-dependent IL-10 induction by OLL2712, as illustrated in Fig. 1c. However, our observations indicate that the TLR2 agonist Pam3CSK4 alone did not induce IL-10 production. There was an unexpected disparity in IL-10 production between OLL2712 and TLR2 agonists. Consequently, we hypothesized that pathways other than TLR2 were involved in OLL2712-induced IL-10 production.

Recent studies have emphasized the capacity of bacteria to activate CLRs, thereby modulating IL-10 production. Notably, co-stimulation of TLR2 with Dectin-1 or Mincle has been reported to robustly induce IL-10 production. For example, Dillon et al. reported that zymosan, a stimulant of both TLR2 and Dectin-1, induced IL-10 production in DCs (20). Patin et al. reported that stimulation with both TLR2 and Mincle strongly induced IL-10 production in bone marrow-derived macrophages (21). Furthermore, LAB induce IL-10 production via CLRs. Indeed, Parad et al. reported that *L. brevis* activates Mincle, which regulates IL-10 production (26). Based on these reports, we considered the involvement of CLR in this mechanism. Our data demonstrate the inhibition of OLL2712-induced IL-10 production by a Syk phosphorylation inhibitor. Subsequent CLR gene knockdown assays provided evidence for the role of Mincle in IL-10 production by OLL2712, implicating the Mincle-Syk pathway in this mechanism. Intriguingly, despite the suppression of OLL2712-induced IL-10 production by Mincle gene knockdown, neutralizing antibody-mediated Mincle blockade failed to elicit comparable suppression. We believe that this anomaly may stem from a potential intracellular interaction between Mincle and OLL2712. Kerscher et al. reported that Mincle can be activated via the MYD88 signal but remains intracellularly localized (27). Nagata et al. reported that intracellularly localized Mincle may interact with β-glucosylceramide in cells (28). Indeed, our data demonstrated that the inhibition of tsDCs phagocytosis concurrently suppressed Syk phosphorylation and IL-10 production, suggesting a potential intracellular interaction between OLL2712 and Mincle.

The identity of the Mincle ligand of OLL2712 poses an intriguing question. Recent reports have proposed that the surface layer (S-layer) proteins of LAB serve as ligands for Mincle, which regulates cytokine production in immune cells. S-layer proteins are conventionally associated with the outermost layers of bacteria. If the S-layer proteins were indeed Mincle ligands for OLL2712, this would seemingly contradict our findings with Mincle-neutralizing antibodies. Imai et al. reported that the LTA anchor portion of the gram-positive bacterial Group A *Streptococcus*, which is a monoglucosyldiacylglycerol, functions as a ligand for Mincle, while intact LTA from the same bacteria showed TLR2 activity but no Mincle activation (29). If the LTA anchor moiety of OLL2712 serves as a Mincle ligand, in other words, under conditions where the Mincle ligand is not exposed to the bacterial surface, this will align with our experimental outcomes. Shah et al. reported that glycolipids of *L. plantarum* could act as a Mincle ligand (30), suggesting that the LTA anchor of OLL2712 may function as a Mincle ligand. In the future, the OLL2712-derived Mincle ligand should be clarified to reinforce this mechanism.

In summary, our study established that OLL2712 activates TLR2 and induces IL-10 production through the subsequent activation of Mincle. We previously identified OLL2712 as a strain with a robust capacity to induce IL-10 production. This study provides vital insights into the underlying factors. However, this mechanism has not yet been fully elucidated, and there may be involvement of other receptors or ligands not addressed in this study. Future investigations building upon this study may serve as a foundation for identifying the determinants that confer OLL2712 with a predominant role in IL-10 induction.

## Data Availability

The data presented in this study are available upon request from the corresponding author.

## SUPPLEMENTAL MATERIAL

Supplemental figures spectrum.01196-24-s0001.docx Figures S1 to S5.

ASM does not own the copyrights to Supplemental Material that may be linked to, or accessed through, an article. The authors have granted ASM a non-exclusive, world-wide license to publish the Supplemental Material files. Please contact the corresponding author directly for reuse.
