# Supplementary material for: Regulation of IL-10 production in dendritic cells is controlled by the co-activation of TLR2 and Mincle by Lactiplantibacillus plantarum OLL2712
Source: Microbiol Spectr. 2025 Feb 4;13(3):e01196-24. doi: 10.1128/spectrum.01196-24 (PMC11878067; doi:10.1128/spectrum.01196-24)
Supplement: Supplemental figures — Figures S1 to S5. [file spectrum.01196-24-s0001.docx]

**Supplemental Materials**


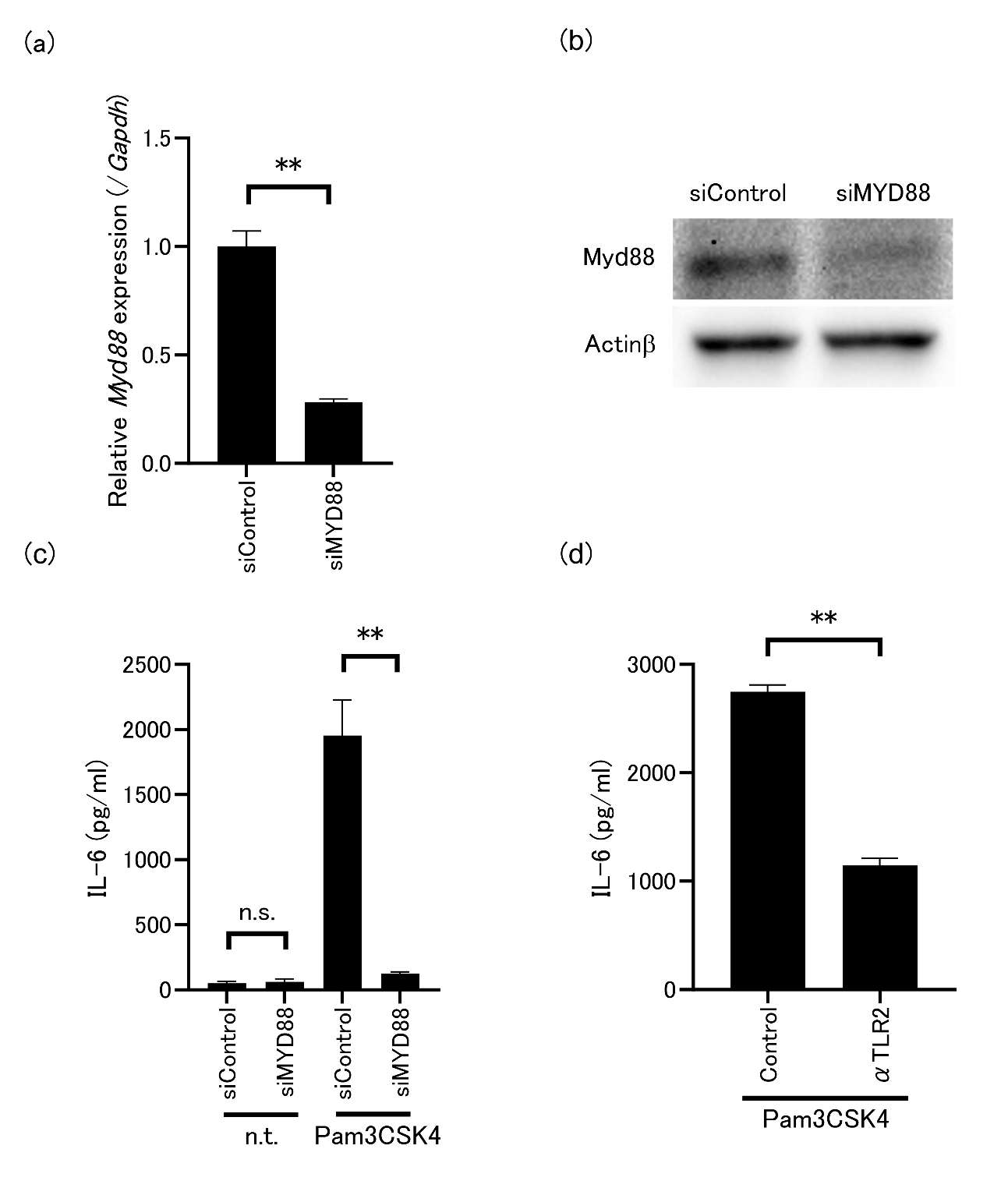


**Figure S1.**

Effect of small interfering (si) RNA for *Myd88* and TLR2 neutralizing antibody. tsDCs were electroporated with siRNA targeting *Myd88* (siMYD88), or control siRNA (siControl), and (**a**) *Myd88* mRNA expression and (**b**) protein levels were measured to confirm knockdown efficiency. (**c**) *Myd88* and control siRNA treated cells were stimulated with Pam3CSK4 (100 ng/mL) for 24 h and IL-6 levels in supernatant was determined. (**d**) Effect of anti-TLR2 antibody on Pam3CSK4 (100 ng/mL)-induced IL-6 levels was determined. Data are shown as the mean ± SD of cultured wells (n = 3). **: *p* < 0.01.

**Figure S2.
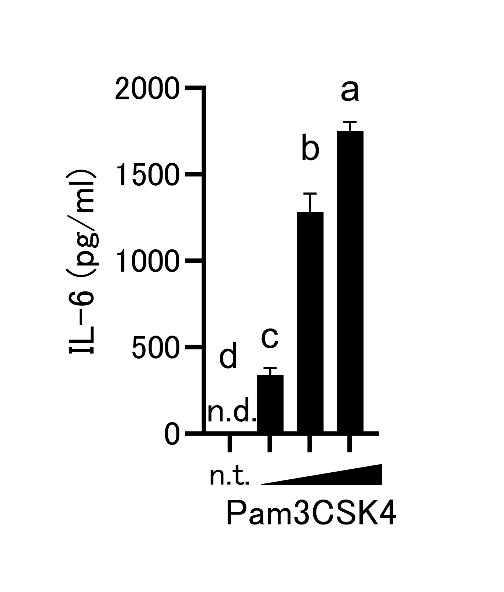
**

Effect of Pam3CSK4 treatment on IL-6 production in tsDCs. tsDCs were cultured with various concentrations of Pam3CSK4 (10, 50, and 100 ng/mL) for 24 h. n.d. indicates not detected. The detection limit for IL-6 using this method was 31.3 µg/mL. Values not sharing a common letter were significantly different (*p* < 0.01). Data are presented as the mean ± SD of the cultured wells (n = 3).


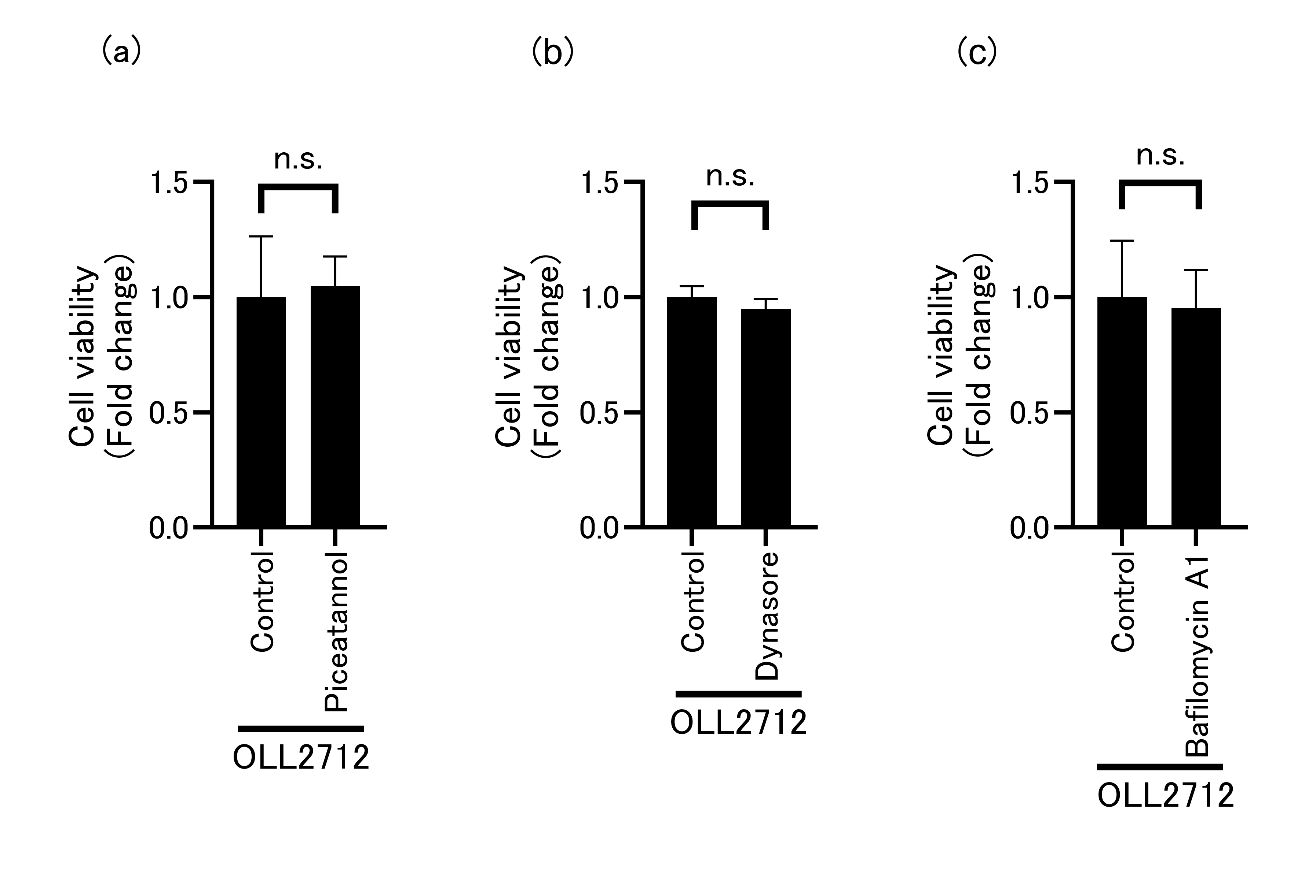


**Figure S3.**

The viability of tsDCs was determined using the WST-1 assay after treatment with (a) piceatannol, (b) dynasore, or (c) bafilomycin A1. n.s. indicates no statistical significance. Data are presented as the mean ± SD of the cultured wells (n = 3).


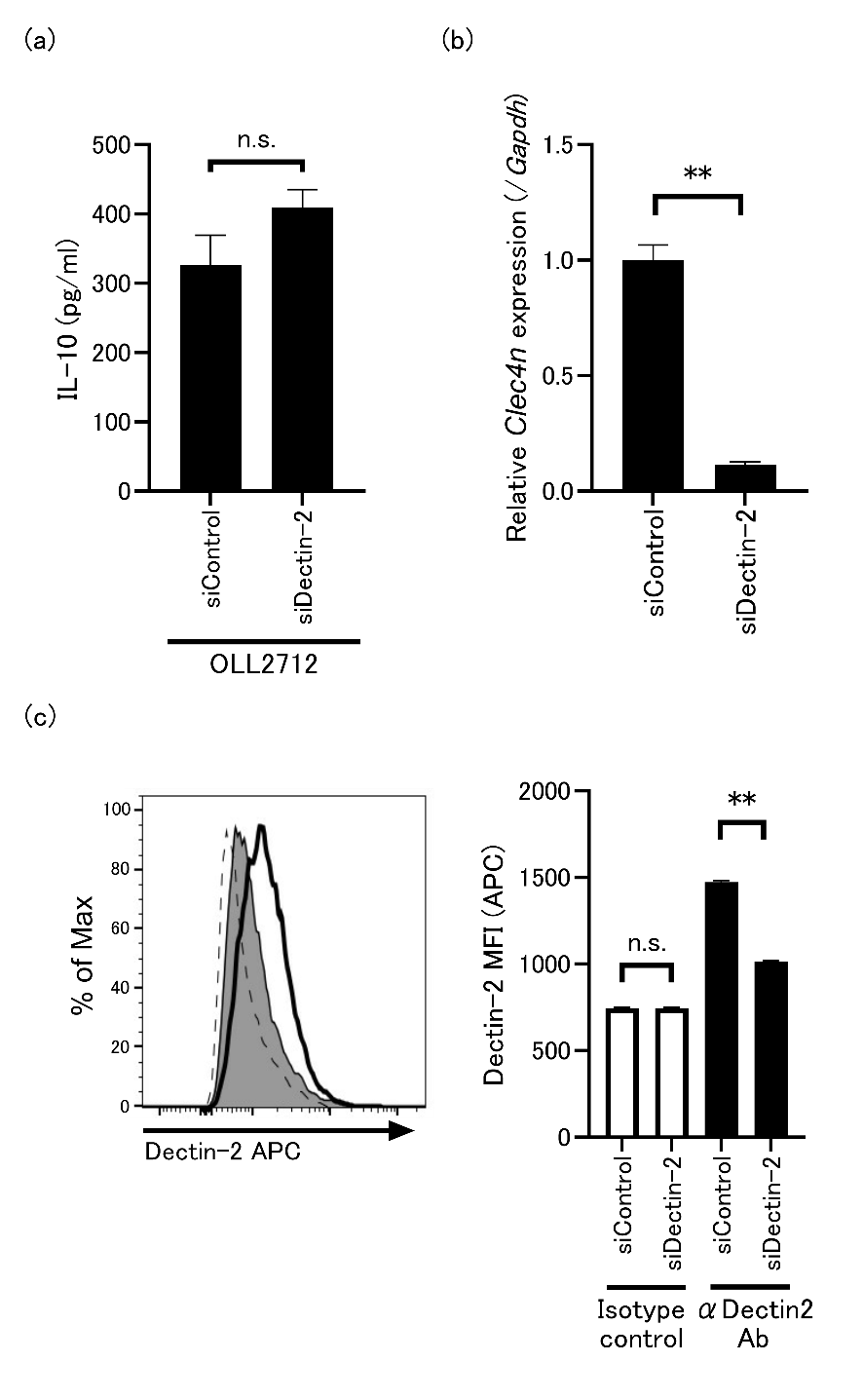


**Figure S4**.

Effect of Dectin-2 gene knockdown by siRNA on OLL2712-induced IL-10 production (**a**). tsDCs were electroporated with siRNA targeting *Clec4n* (siDectin-2) or control siRNA (siControl), followed by OLL2712 stimulation. The effect of *Clec4n* siRNA is shown by (**b**) *Clec4n* mRNA expression level, as well as (**c**) Dectin-2 surface expression level by FACS analysis (Isotype control stained; dashed line, *Clec4n* siRNA treated cells; filled histogram, control siRNA treated cells; bold solid line), and their mean fluorescence intensity (MFI). n.s. indicates no statistical significance. Data are presented as the mean ± SD of the cultured wells (n = 3). **: *p* < 0.01.


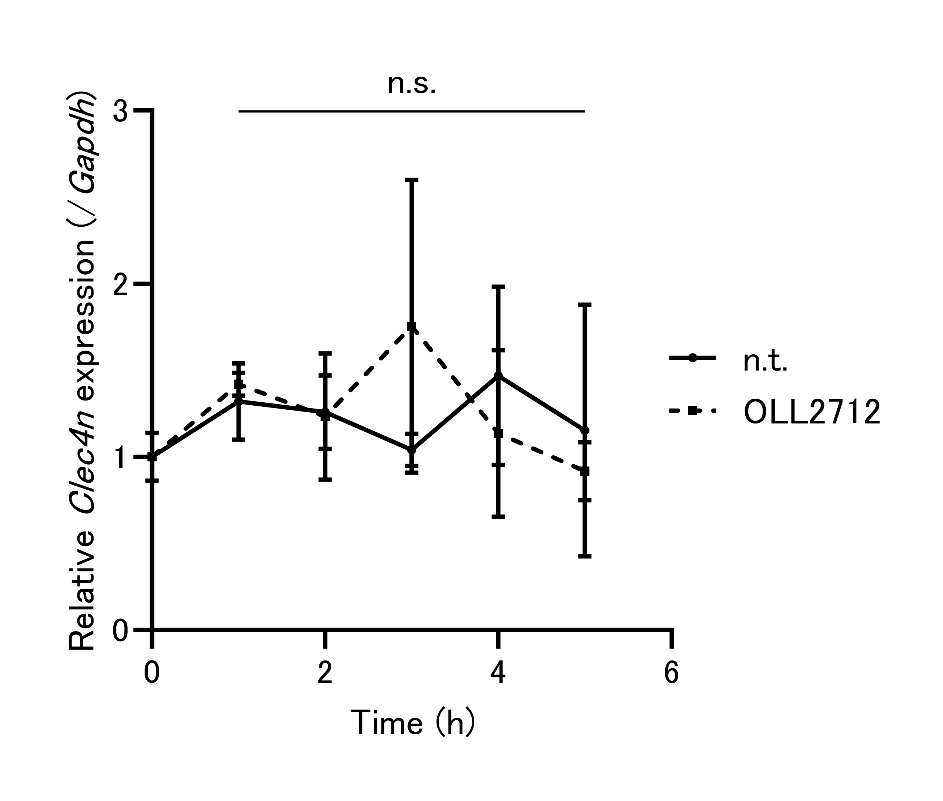


**Figure S5.**

*Clec4n* expression in tsDCs stimulated with OLL2712. Relative gene expression levels were normalized to Glyceraldegyde-3-phosphate dehydrogenase gene (*Gapdh*). n.t. indicates not treated for tsDCs. n.s. indicates no statistical significance. Data are presented as the mean ± SD of the cultured wells (n = 3).
